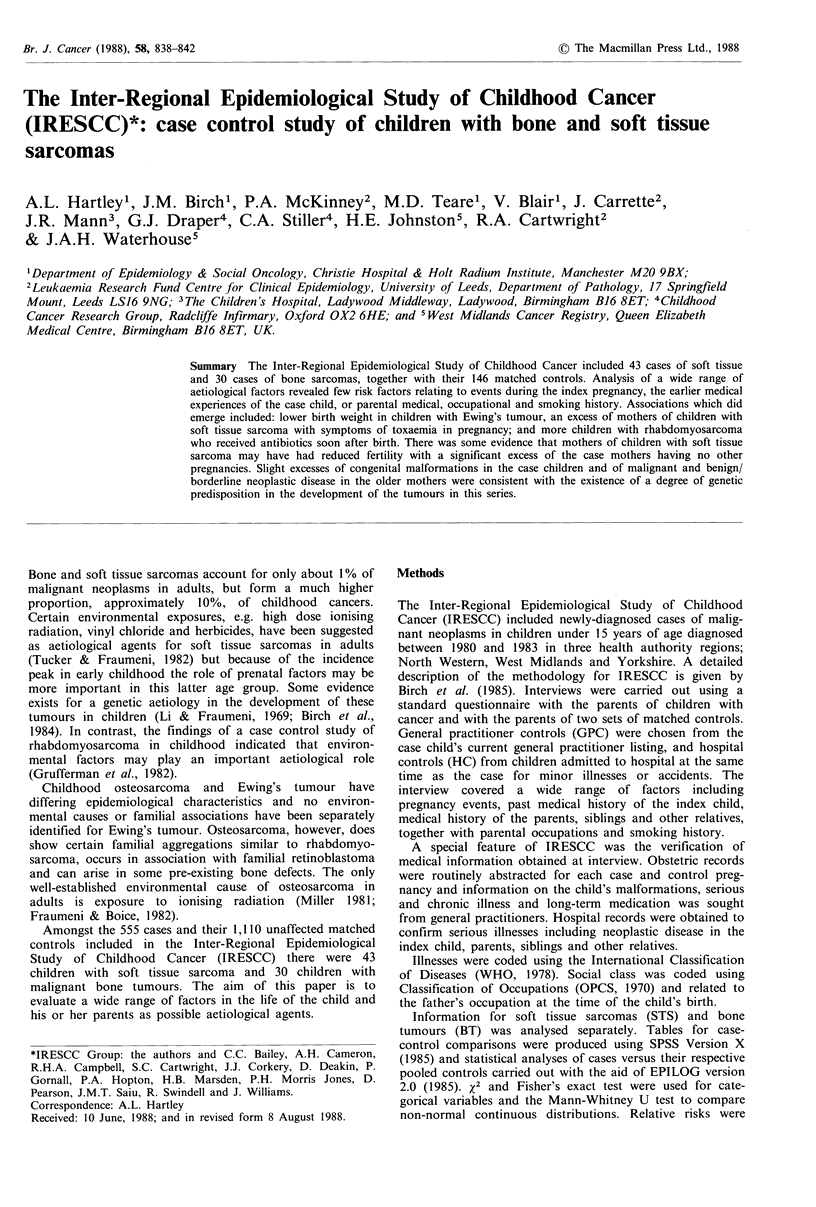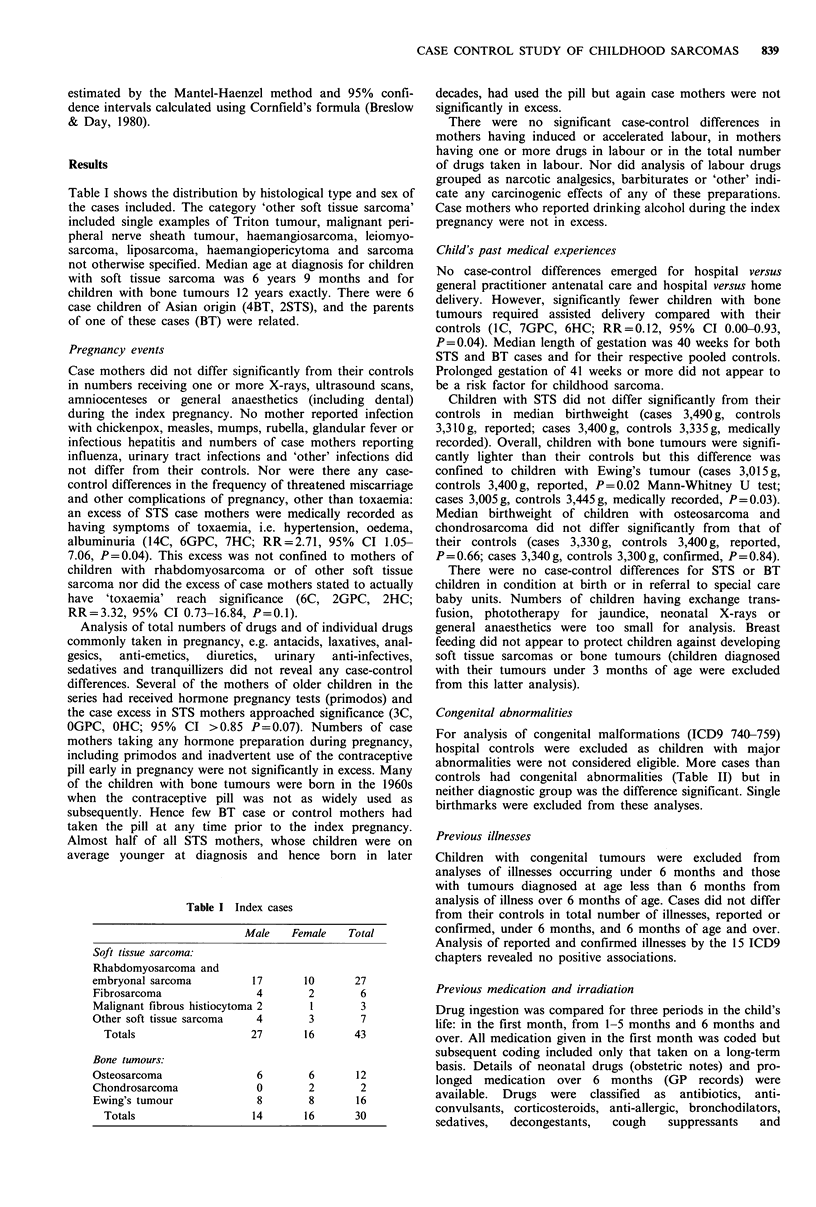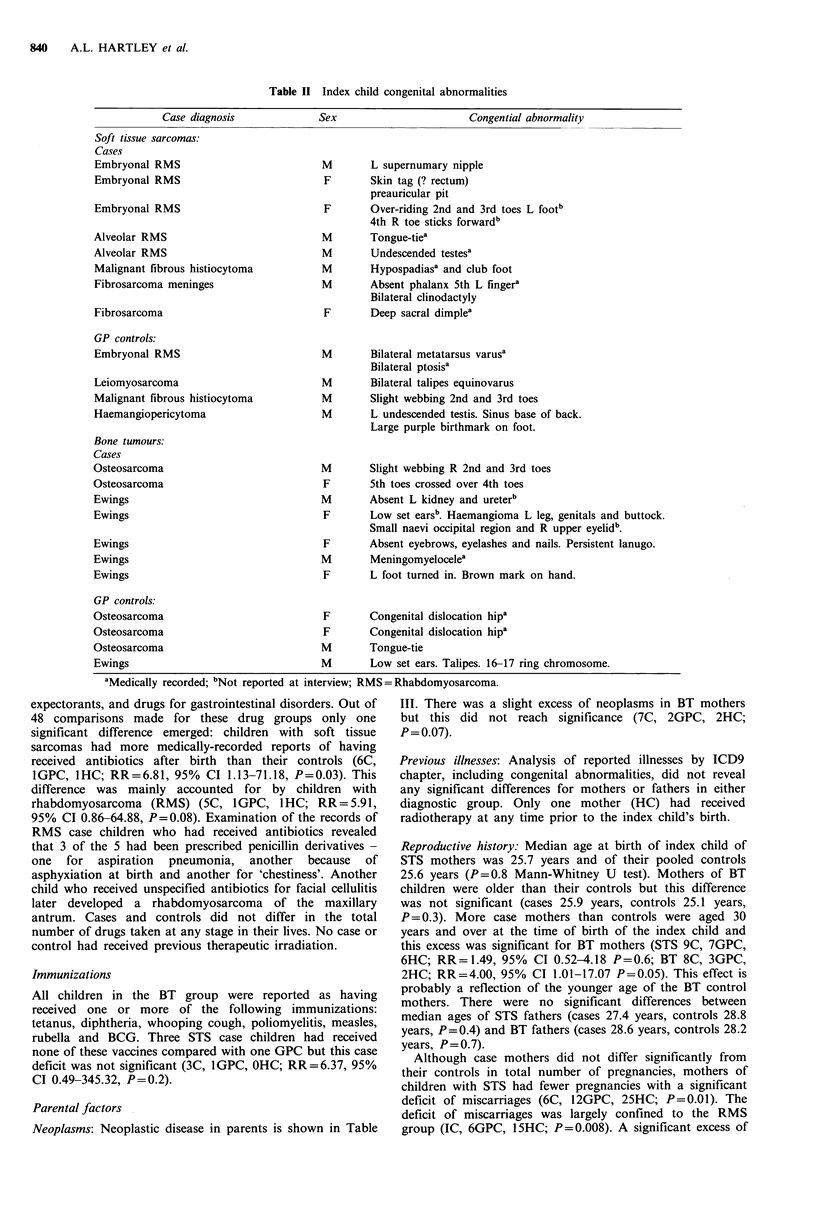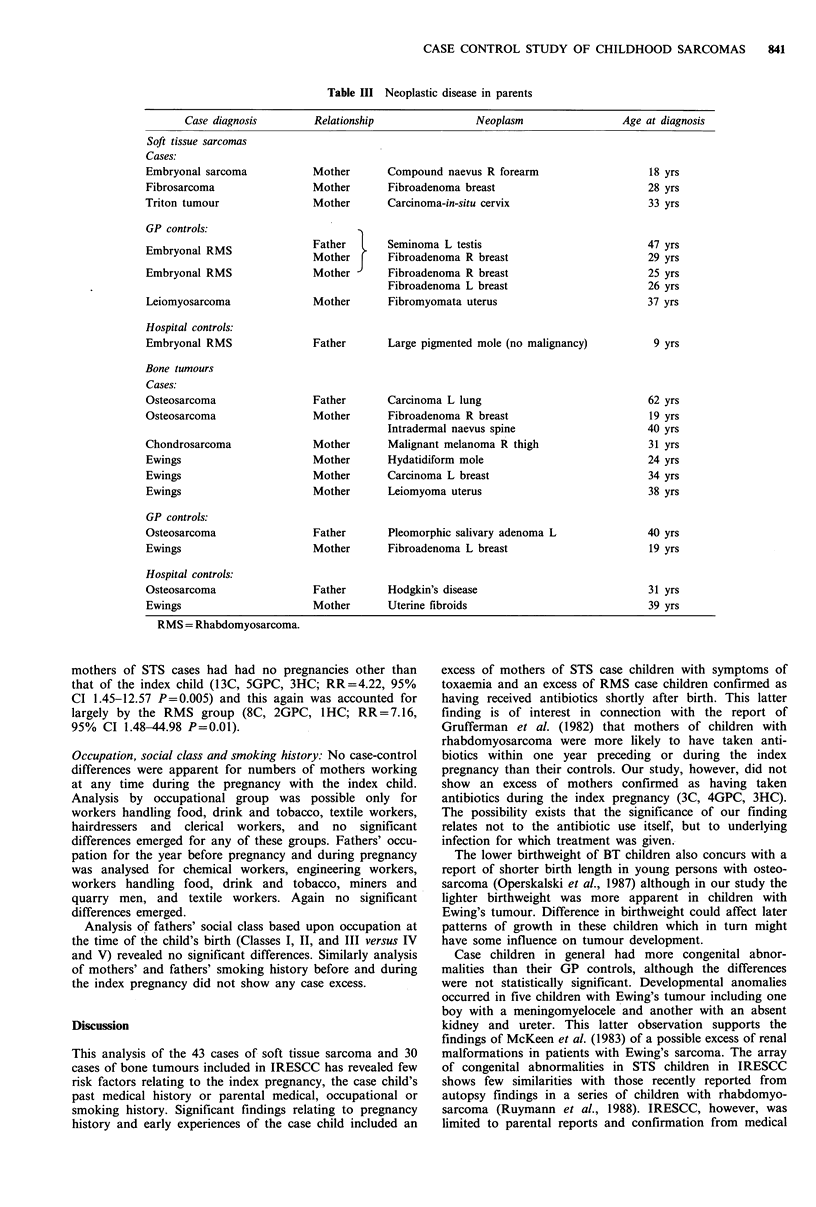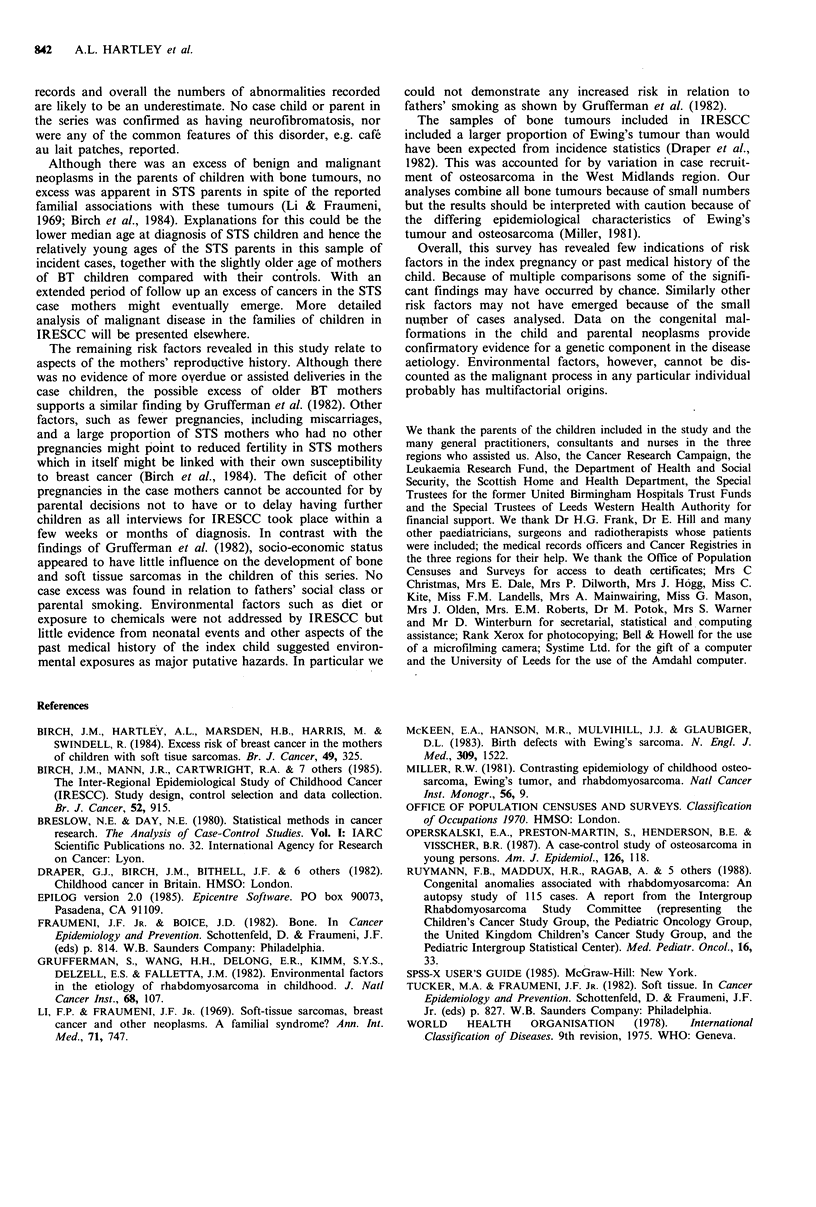# The Inter-Regional Epidemiological Study of Childhood Cancer (IRESCC): case control study of children with bone and soft tissue sarcomas.

**DOI:** 10.1038/bjc.1988.321

**Published:** 1988-12

**Authors:** A. L. Hartley, J. M. Birch, P. A. McKinney, M. D. Teare, V. Blair, J. Carrette, J. R. Mann, G. J. Draper, C. A. Stiller, H. E. Johnston

**Affiliations:** Department of Epidemiology & Social Oncology, Christie Hospital & Holt Radium Institute, Manchester, UK.

## Abstract

The Inter-Regional Epidemiological Study of Childhood Cancer included 43 cases of soft tissue and 30 cases of bone sarcomas, together with their 146 matched controls. Analysis of a wide range of aetiological factors revealed few risk factors relating to events during the index pregnancy, the earlier medical experiences of the case child, or parental medical, occupational and smoking history. Associations which did emerge included: lower birth weight in children with Ewing's tumour, an excess of mothers of children with soft tissue sarcoma with symptoms of toxaemia in pregnancy; and more children with rhabdomyosarcoma who received antibiotics soon after birth. There was some evidence that mothers of children with soft tissue sarcoma may have had reduced fertility with a significant excess of the case mothers having no other pregnancies. Slight excesses of congenital malformations in the case children and of malignant and benign/borderline neoplastic disease in the older mothers were consistent with the existence of a degree of genetic predisposition in the development of the tumours in this series.


Br. J. Cancer (1988), 58, 838–842 © The Macmillan Press Ltd., 1988

### The Inter-Regional Epidemiological Study of Childhood Cancer (IRESCC)*: case control study of children with bone and soft tissue sarcomas

A.L. Hartley[1], J.M. Birch[1], P.A. McKinney[2], M.D. Teare[1], V. Blair[1], J. Carrette[2], J.R. Mann[3], G.J. Draper[4], C.A. Stiller[4], H.E. Johnston[5], R.A. Cartwright[2] & J.A.H. Waterhouse[5]

[1]*Department of Epidemiology & Social Oncology, Christie Hospital & Holt Radium Institute, Manchester M20 9BX;* [2]*Leukaemia Research Fund Centre for Clinical Epidemiology, University of Leeds, Department of Pathology, 17 Springfield Mount, Leeds LS16 9NG;* [3]*The Children's Hospital, Ladywood Middleway, Ladywood, Birmingham B16 8ET;* [4]*Childhood Cancer Research Group, Radcliffe Infirmary, Oxford OX2 6HE; and* [5]*West Midlands Cancer Registry, Queen Elizabeth Medical Centre, Birmingham B16 8ET, UK.*

**Summary** The Inter-Regional Epidemiological Study of Childhood Cancer included 43 cases of soft tissue and 30 cases of bone sarcomas, together with their 146 matched controls. Analysis of a wide range of aetiological factors revealed few risk factors relating to events during the index pregnancy, the earlier medical experiences of the case child, or parental medical, occupational and smoking history. Associations which did emerge included: lower birth weight in children with Ewing's tumour, an excess of mothers of children with soft tissue sarcoma with symptoms of toxaemia in pregnancy; and more children with rhabdomyosarcoma who received antibiotics soon after birth. There was some evidence that mothers of children with soft tissue sarcoma may have had reduced fertility with a significant excess of the case mothers having no other pregnancies. Slight excesses of congenital malformations in the case children and of malignant and benign/borderline neoplastic disease in the older mothers were consistent with the existence of a degree of genetic predisposition in the development of the tumours in this series.

Bone and soft tissue sarcomas account for only about 1% of malignant neoplasms in adults, but form a much higher proportion, approximately 10%, of childhood cancers. Certain environmental exposures, e.g. high dose ionising radiation, vinyl chloride and herbicides, have been suggested as aetiological agents for soft tissue sarcomas in adults (Tucker & Fraumeni, 1982) but because of the incidence peak in early childhood the role of prenatal factors may be more important in this latter age group. Some evidence exists for a genetic aetiology in the development of these tumours in children (Li & Fraumeni, 1969; Birch *et al.*, 1984). In contrast, the findings of a case control study of rhabdomyosarcoma in childhood indicated that environmental factors may play an important aetiological role (Grufferman *et al.*, 1982).

Childhood osteosarcoma and Ewing's tumour have differing epidemiological characteristics and no environmental causes or familial associations have been separately identified for Ewing's tumour. Osteosarcoma, however, does show certain familial aggregations similar to rhabdomyosarcoma, occurs in association with familial retinoblastoma and can arise in some pre-existing bone defects. The only well-established environmental cause of osteosarcoma in adults is exposure to ionising radiation (Miller 1981; Fraumeni & Boice, 1982).

Amongst the 555 cases and their 1,110 unaffected matched controls included in the Inter-Regional Epidemiological Study of Childhood Cancer (IRESCC) there were 43 children with soft tissue sarcoma and 30 children with malignant bone tumours. The aim of this paper is to evaluate a wide range of factors in the life of the child and his or her parents as possible aetiological agents.

*IRESCC Group: the authors and C.C. Bailey, A.H. Cameron, R.H.A. Campbell, S.C. Cartwright, J.J. Corkery, D. Deakin, P. Gornall, P.A. Hopton, H.B. Marsden, P.H. Morris Jones, D. Pearson, J.M.T. Saiu, R. Swindell and J. Williams.

Correspondence: A.L. Hartley

Received: 10 June, 1988; and in revised form 8 August 1988.

### Methods

The Inter-Regional Epidemiological Study of Childhood Cancer (IRESCC) included newly-diagnosed cases of malignant neoplasms in children under 15 years of age diagnosed between 1980 and 1983 in three health authority regions; North Western, West Midlands and Yorkshire. A detailed description of the methodology for IRESCC is given by Birch *et al.* (1985). Interviews were carried out using a standard questionnaire with the parents of children with cancer and with the parents of two sets of matched controls. General practitioner controls (GPC) were chosen from the case child's current general practitioner listing, and hospital controls (HC) from children admitted to hospital at the same time as the case for minor illnesses or accidents. The interview covered a wide range of factors including pregnancy events, past medical history of the index child, medical history of the parents, siblings and other relatives, together with parental occupations and smoking history.

A special feature of IRESCC was the verification of medical information obtained at interview. Obstetric records were routinely abstracted for each case and control pregnancy and information on the child's malformations, serious and chronic illness and long-term medication was sought from general practitioners. Hospital records were obtained to confirm serious illnesses including neoplastic disease in the index child, parents, siblings and other relatives.

Illnesses were coded using the International Classification of Diseases (WHO, 1978). Social class was coded using Classification of Occupations (OPCS, 1970) and related to the father's occupation at the time of the child's birth.

Information for soft tissue sarcomas (STS) and bone tumours (BT) was analysed separately. Tables for case-control comparisons were produced using SPSS Version X (1985) and statistical analyses of cases versus their respective pooled controls carried out with the aid of EPILOG version 2.0 (1985). $\chi^2$ and Fisher's exact test were used for categorical variables and the Mann-Whitney U test to compare non-normal continuous distributions. Relative risks were estimated by the Mantel-Haenzel method and 95% confidence intervals calculated using Cornfield's formula (Breslow & Day, 1980).

### Results

Table I shows the distribution by histological type and sex of the cases included. The category 'other soft tissue sarcoma' included single examples of Triton tumour, malignant peripheral nerve sheath tumour, haemangiosarcoma, leiomyosarcoma, liposarcoma, haemangiopericytoma and sarcoma not otherwise specified. Median age at diagnosis for children with soft tissue sarcoma was 6 years 9 months and for children with bone tumours 12 years exactly. There were 6 case children of Asian origin (4BT, 2STS), and the parents of one of these cases (BT) were related.

### *Pregnancy events*

Case mothers did not differ significantly from their controls in numbers receiving one or more X-rays, ultrasound scans, amniocenteses or general anaesthetics (including dental) during the index pregnancy. No mother reported infection with chickenpox, measles, mumps, rubella, glandular fever or infectious hepatitis and numbers of case mothers reporting influenza, urinary tract infections and 'other' infections did not differ from their controls. Nor were there any case-control differences in the frequency of threatened miscarriage and other complications of pregnancy, other than toxaemia: an excess of STS case mothers were medically recorded as having symptoms of toxaemia, i.e. hypertension, oedema, albuminuria (14C, 6GPC, 7HC; RR=2.71, 95% CI 1.05–7.06, $P=0.04$). This excess was not confined to mothers of children with rhabdomyosarcoma or of other soft tissue sarcoma nor did the excess of case mothers stated to actually have 'toxaemia' reach significance (6C, 2GPC, 2HC; RR=3.32, 95% CI 0.73–16.84, $P=0.1$).

Analysis of total numbers of drugs and of individual drugs commonly taken in pregnancy, e.g. antacids, laxatives, analgesics, anti-emetics, diuretics, urinary anti-infectives, sedatives and tranquillizers did not reveal any case-control differences. Several of the mothers of older children in the series had received hormone pregnancy tests (primodos) and the case excess in STS mothers approached significance (3C, 0GPC, 0HC; 95% CI >0.85 $P=0.07$). Numbers of case mothers taking any hormone preparation during pregnancy, including primodos and inadvertent use of the contraceptive pill early in pregnancy were not significantly in excess. Many of the children with bone tumours were born in the 1960s when the contraceptive pill was not as widely used as subsequently. Hence few BT case or control mothers had taken the pill at any time prior to the index pregnancy. Almost half of all STS mothers, whose children were on average younger at diagnosis and hence born in later decades, had used the pill but again case mothers were not significantly in excess.

There were no significant case-control differences in mothers having induced or accelerated labour, in mothers having one or more drugs in labour or in the total number of drugs taken in labour. Nor did analysis of labour drugs grouped as narcotic analgesics, barbiturates or 'other' indicate any carcinogenic effects of any of these preparations. Case mothers who reported drinking alcohol during the index pregnancy were not in excess.

**Table I** Index cases

| | *Male* | *Female* | *Total* |
|---|---|---|---|
| *Soft tissue sarcoma:* | | | |
| Rhabdomyosarcoma and embryonal sarcoma | 17 | 10 | 27 |
| Fibrosarcoma | 4 | 2 | 6 |
| Malignant fibrous histiocytoma | 2 | 1 | 3 |
| Other soft tissue sarcoma | 4 | 3 | 7 |
| Totals | 27 | 16 | 43 |
| *Bone tumours:* | | | |
| Osteosarcoma | 6 | 6 | 12 |
| Chondrosarcoma | 0 | 2 | 2 |
| Ewing's tumour | 8 | 8 | 16 |
| Totals | 14 | 16 | 30 |

### *Child's past medical experiences*

No case-control differences emerged for hospital *versus* general practitioner antenatal care and hospital *versus* home delivery. However, significantly fewer children with bone tumours required assisted delivery compared with their controls (1C, 7GPC, 6HC; RR=0.12, 95% CI 0.00–0.93, $P=0.04$). Median length of gestation was 40 weeks for both STS and BT cases and for their respective pooled controls. Prolonged gestation of 41 weeks or more did not appear to be a risk factor for childhood sarcoma.

Children with STS did not differ significantly from their controls in median birthweight (cases 3,490 g, controls 3,310 g, reported; cases 3,400 g, controls 3,335 g, medically recorded). Overall, children with bone tumours were significantly lighter than their controls but this difference was confined to children with Ewing's tumour (cases 3,015 g, controls 3,400 g, reported, $P=0.02$ Mann-Whitney U test; cases 3,005 g, controls 3,445 g, medically recorded, $P=0.03$). Median birthweight of children with osteosarcoma and chondrosarcoma did not differ significantly from that of their controls (cases 3,330 g, controls 3,400 g, reported, $P=0.66$; cases 3,340 g, controls 3,300 g, confirmed, $P=0.84$).

There were no case-control differences for STS or BT children in condition at birth or in referral to special care baby units. Numbers of children having exchange transfusion, phototherapy for jaundice, neonatal X-rays or general anaesthetics were too small for analysis. Breast feeding did not appear to protect children against developing soft tissue sarcomas or bone tumours (children diagnosed with their tumours under 3 months of age were excluded from this latter analysis).

### *Congenital abnormalities*

For analysis of congenital malformations (ICD9 740–759) hospital controls were excluded as children with major abnormalities were not considered eligible. More cases than controls had congenital abnormalities (Table II) but in neither diagnostic group was the difference significant. Single birthmarks were excluded from these analyses.

### *Previous illnesses*

Children with congenital tumours were excluded from analyses of illnesses occurring under 6 months and those with tumours diagnosed at age less than 6 months from analysis of illness over 6 months of age. Cases did not differ from their controls in total number of illnesses, reported or confirmed, under 6 months, and 6 months of age and over. Analysis of reported and confirmed illnesses by the 15 ICD9 chapters revealed no positive associations.

### *Previous medication and irradiation*

Drug ingestion was compared for three periods in the child's life: in the first month, from 1–5 months and 6 months and over. All medication given in the first month was coded but subsequent coding included only that taken on a long-term basis. Details of neonatal drugs (obstetric notes) and prolonged medication over 6 months (GP records) were available. Drugs were classified as antibiotics, anticonvulsants, corticosteroids, anti-allergic, bronchodilators, sedatives, decongestants, cough suppressants and

**Table II** Index child congenital abnormalities

| *Case diagnosis* | *Sex* | *Congential abnormality* |
|---|---|---|
| *Soft tissue sarcomas:* | | |
| *Cases* | | |
| Embryonal RMS | M | L supernumary nipple |
| Embryonal RMS | F | Skin tag (? rectum)<br>preauricular pit |
| Embryonal RMS | F | Over-riding 2nd and 3rd toes L foot[b]<br>4th R toe sticks forward[b] |
| Alveolar RMS | M | Tongue-tie[a] |
| Alveolar RMS | M | Undescended testes[a] |
| Malignant fibrous histiocytoma | M | Hypospadias[a] and club foot |
| Fibrosarcoma meninges | M | Absent phalanx 5th L finger[a]<br>Bilateral clinodactyly |
| Fibrosarcoma | F | Deep sacral dimple[a] |
| *GP controls:* | | |
| Embryonal RMS | M | Bilateral metatarsus varus[a]<br>Bilateral ptosis[a] |
| Leiomyosarcoma | M | Bilateral talipes equinovarus |
| Malignant fibrous histiocytoma | M | Slight webbing 2nd and 3rd toes |
| Haemangiopericytoma | M | L undescended testis. Sinus base of back.<br>Large purple birthmark on foot. |
| *Bone tumours:* | | |
| *Cases* | | |
| Osteosarcoma | M | Slight webbing R 2nd and 3rd toes |
| Osteosarcoma | F | 5th toes crossed over 4th toes |
| Ewings | M | Absent L kidney and ureter[b] |
| Ewings | F | Low set ears[b]. Haemangioma L leg, genitals and buttock.<br>Small naevi occipital region and R upper eyelid[b]. |
| Ewings | F | Absent eyebrows, eyelashes and nails. Persistent lanugo. |
| Ewings | M | Meningomyelocele[a] |
| Ewings | F | L foot turned in. Brown mark on hand. |
| *GP controls:* | | |
| Osteosarcoma | F | Congenital dislocation hip[a] |
| Osteosarcoma | F | Congenital dislocation hip[a] |
| Osteosarcoma | M | Tongue-tie |
| Ewings | M | Low set ears. Talipes. 16–17 ring chromosome. |

[a]Medically recorded; [b]Not reported at interview; RMS = Rhabdomyosarcoma.

expectorants, and drugs for gastrointestinal disorders. Out of 48 comparisons made for these drug groups only one significant difference emerged: children with soft tissue sarcomas had more medically-recorded reports of having received antibiotics after birth than their controls (6C, 1GPC, 1HC; RR = 6.81, 95% CI 1.13–71.18, $P = 0.03$). This difference was mainly accounted for by children with rhabdomyosarcoma (RMS) (5C, 1GPC, 1HC; RR = 5.91, 95% CI 0.86–64.88, $P = 0.08$). Examination of the records of RMS case children who had received antibiotics revealed that 3 of the 5 had been prescribed penicillin derivatives – one for aspiration pneumonia, another because of asphyxiation at birth and another for 'chestiness'. Another child who received unspecified antibiotics for facial cellulitis later developed a rhabdomyosarcoma of the maxillary antrum. Cases and controls did not differ in the total number of drugs taken at any stage in their lives. No case or control had received previous therapeutic irradiation.

### *Immunizations*

All children in the BT group were reported as having received one or more of the following immunizations: tetanus, diphtheria, whooping cough, poliomyelitis, measles, rubella and BCG. Three STS case children had received none of these vaccines compared with one GPC but this case deficit was not significant (3C, 1GPC, 0HC; RR = 6.37, 95% CI 0.49–345.32, $P = 0.2$).

### *Parental factors*

*Neoplasms*: Neoplastic disease in parents is shown in Table III. There was a slight excess of neoplasms in BT mothers but this did not reach significance (7C, 2GPC, 2HC; $P = 0.07$).

*Previous illnesses*: Analysis of reported illnesses by ICD9 chapter, including congenital abnormalities, did not reveal any significant differences for mothers or fathers in either diagnostic group. Only one mother (HC) had received radiotherapy at any time prior to the index child's birth.

*Reproductive history:* Median age at birth of index child of STS mothers was 25.7 years and of their pooled controls 25.6 years ($P = 0.8$ Mann-Whitney U test). Mothers of BT children were older than their controls but this difference was not significant (cases 25.9 years, controls 25.1 years, $P = 0.3$). More case mothers than controls were aged 30 years and over at the time of birth of the index child and this excess was significant for BT mothers (STS 9C, 7GPC, 6HC; RR = 1.49, 95% CI 0.52–4.18 $P = 0.6$; BT 8C, 3GPC, 2HC; RR = 4.00, 95% CI 1.01–17.07 $P = 0.05$). This effect is probably a reflection of the younger age of the BT control mothers. There were no significant differences between median ages of STS fathers (cases 27.4 years, controls 28.8 years, $P = 0.4$) and BT fathers (cases 28.6 years, controls 28.2 years, $P = 0.7$).

Although case mothers did not differ significantly from their controls in total number of pregnancies, mothers of children with STS had fewer pregnancies with a significant deficit of miscarriages (6C, 12GPC, 25HC; $P = 0.01$). The deficit of miscarriages was largely confined to the RMS group (1C, 6GPC, 15HC; $P = 0.008$). A significant excess of

**Table III** Neoplastic disease in parents

| *Case diagnosis* | *Relationship* | *Neoplasm* | *Age at diagnosis* |
|---|---|---|---|
| *Soft tissue sarcomas* | | | |
| *Cases:* | | | |
| Embryonal sarcoma | Mother | Compound naevus R forearm | 18 yrs |
| Fibrosarcoma | Mother | Fibroadenoma breast | 28 yrs |
| Triton tumour | Mother | Carcinoma-*in-situ* cervix | 33 yrs |
| *GP controls:* | | | |
| Embryonal RMS | Father | Seminoma L testis | 47 yrs |
| | Mother | Fibroadenoma R breast | 29 yrs |
| Embryonal RMS | Mother | Fibroadenoma R breast | 25 yrs |
| | | Fibroadenoma L breast | 26 yrs |
| Leiomyosarcoma | Mother | Fibromyomata uterus | 37 yrs |
| *Hospital controls:* | | | |
| Embryonal RMS | Father | Large pigmented mole (no malignancy) | 9 yrs |
| *Bone tumours* | | | |
| *Cases:* | | | |
| Osteosarcoma | Father | Carcinoma L lung | 62 yrs |
| Osteosarcoma | Mother | Fibroadenoma R breast | 19 yrs |
| | | Intradermal naevus spine | 40 yrs |
| Chondrosarcoma | Mother | Malignant melanoma R thigh | 31 yrs |
| Ewings | Mother | Hydatidiform mole | 24 yrs |
| Ewings | Mother | Carcinoma L breast | 34 yrs |
| Ewings | Mother | Leiomyoma uterus | 38 yrs |
| *GP controls:* | | | |
| Osteosarcoma | Father | Pleomorphic salivary adenoma L | 40 yrs |
| Ewings | Mother | Fibroadenoma L breast | 19 yrs |
| *Hospital controls:* | | | |
| Osteosarcoma | Father | Hodgkin's disease | 31 yrs |
| Ewings | Mother | Uterine fibroids | 39 yrs |

RMS = Rhabdomyosarcoma.

mothers of STS cases had had no pregnancies other than that of the index child (13C, 5GPC, 3HC; RR = 4.22, 95% CI 1.45–12.57 $P = 0.005$) and this again was accounted for largely by the RMS group (8C, 2GPC, 1HC; RR = 7.16, 95% CI 1.48–44.98 $P = 0.01$).

*Occupation, social class and smoking history:* No case-control differences were apparent for numbers of mothers working at any time during the pregnancy with the index child. Analysis by occupational group was possible only for workers handling food, drink and tobacco, textile workers, hairdressers and clerical workers, and no significant differences emerged for any of these groups. Fathers' occupation for the year before pregnancy and during pregnancy was analysed for chemical workers, engineering workers, workers handling food, drink and tobacco, miners and quarry men, and textile workers. Again no significant differences emerged.

Analysis of fathers' social class based upon occupation at the time of the child's birth (Classes I, II, and III *versus* IV and V) revealed no significant differences. Similarly analysis of mothers' and fathers' smoking history before and during the index pregnancy did not show any case excess.

### Discussion

This analysis of the 43 cases of soft tissue sarcoma and 30 cases of bone tumours included in IRESCC has revealed few risk factors relating to the index pregnancy, the case child's past medical history or parental medical, occupational or smoking history. Significant findings relating to pregnancy history and early experiences of the case child included an excess of mothers of STS case children with symptoms of toxaemia and an excess of RMS case children confirmed as having received antibiotics shortly after birth. This latter finding is of interest in connection with the report of Grufferman *et al.* (1982) that mothers of children with rhabdomyosarcoma were more likely to have taken antibiotics within one year preceding or during the index pregnancy than their controls. Our study, however, did not show an excess of mothers confirmed as having taken antibiotics during the index pregnancy (3C, 4GPC, 3HC). The possibility exists that the significance of our finding relates not to the antibiotic use itself, but to underlying infection for which treatment was given.

The lower birthweight of BT children also concurs with a report of shorter birth length in young persons with osteosarcoma (Operskalski *et al.*, 1987) although in our study the lighter birthweight was more apparent in children with Ewing's tumour. Difference in birthweight could affect later patterns of growth in these children which in turn might have some influence on tumour development.

Case children in general had more congenital abnormalities than their GP controls, although the differences were not statistically significant. Developmental anomalies occurred in five children with Ewing's tumour including one boy with a meningomyelocele and another with an absent kidney and ureter. This latter observation supports the findings of McKeen *et al.* (1983) of a possible excess of renal malformations in patients with Ewing's sarcoma. The array of congenital abnormalities in STS children in IRESCC shows few similarities with those recently reported from autopsy findings in a series of children with rhabdomyosarcoma (Ruymann *et al.*, 1988). IRESCC, however, was limited to parental reports and confirmation from medical records and overall the numbers of abnormalities recorded are likely to be an underestimate. No case child or parent in the series was confirmed as having neurofibromatosis, nor were any of the common features of this disorder, e.g. café au lait patches, reported.

Although there was an excess of benign and malignant neoplasms in the parents of children with bone tumours, no excess was apparent in STS parents in spite of the reported familial associations with these tumours (Li & Fraumeni, 1969; Birch *et al.*, 1984). Explanations for this could be the lower median age at diagnosis of STS children and hence the relatively young ages of the STS parents in this sample of incident cases, together with the slightly older age of mothers of BT children compared with their controls. With an extended period of follow up an excess of cancers in the STS case mothers might eventually emerge. More detailed analysis of malignant disease in the families of children in IRESCC will be presented elsewhere.

The remaining risk factors revealed in this study relate to aspects of the mothers' reproductive history. Although there was no evidence of more overdue or assisted deliveries in the case children, the possible excess of older BT mothers supports a similar finding by Grufferman *et al.* (1982). Other factors, such as fewer pregnancies, including miscarriages, and a large proportion of STS mothers who had no other pregnancies might point to reduced fertility in STS mothers which in itself might be linked with their own susceptibility to breast cancer (Birch *et al.*, 1984). The deficit of other pregnancies in the case mothers cannot be accounted for by parental decisions not to have or to delay having further children as all interviews for IRESCC took place within a few weeks or months of diagnosis. In contrast with the findings of Grufferman *et al.* (1982), socio-economic status appeared to have little influence on the development of bone and soft tissue sarcomas in the children of this series. No case excess was found in relation to fathers' social class or parental smoking. Environmental factors such as diet or exposure to chemicals were not addressed by IRESCC but little evidence from neonatal events and other aspects of the past medical history of the index child suggested environmental exposures as major putative hazards. In particular we could not demonstrate any increased risk in relation to fathers' smoking as shown by Grufferman *et al.* (1982).

The samples of bone tumours included in IRESCC included a larger proportion of Ewing's tumour than would have been expected from incidence statistics (Draper *et al.*, 1982). This was accounted for by variation in case recruitment of osteosarcoma in the West Midlands region. Our analyses combine all bone tumours because of small numbers but the results should be interpreted with caution because of the differing epidemiological characteristics of Ewing's tumour and osteosarcoma (Miller, 1981).

Overall, this survey has revealed few indications of risk factors in the index pregnancy or past medical history of the child. Because of multiple comparisons some of the significant findings may have occurred by chance. Similarly other risk factors may not have emerged because of the small number of cases analysed. Data on the congenital malformations in the child and parental neoplasms provide confirmatory evidence for a genetic component in the disease aetiology. Environmental factors, however, cannot be discounted as the malignant process in any particular individual probably has multifactorial origins.

We thank the parents of the children included in the study and the many general practitioners, consultants and nurses in the three regions who assisted us. Also, the Cancer Research Campaign, the Leukaemia Research Fund, the Department of Health and Social Security, the Scottish Home and Health Department, the Special Trustees for the former United Birmingham Hospitals Trust Funds and the Special Trustees of Leeds Western Health Authority for financial support. We thank Dr H.G. Frank, Dr E. Hill and many other paediatricians, surgeons and radiotherapists whose patients were included; the medical records officers and Cancer Registries in the three regions for their help. We thank the Office of Population Censuses and Surveys for access to death certificates; Mrs C Christmas, Mrs E. Dale, Mrs P. Dilworth, Mrs J. Hogg, Miss C. Kite, Miss F.M. Landells, Mrs A. Mainwairing, Miss G. Mason, Mrs J. Olden, Mrs. E.M. Roberts, Dr M. Potok, Mrs S. Warner and Mr D. Winterburn for secretarial, statistical and computing assistance; Rank Xerox for photocopying; Bell & Howell for the use of a microfilming camera; Systime Ltd. for the gift of a computer and the University of Leeds for the use of the Amdahl computer.

### References

BIRCH, J.M., HARTLEY, A.L., MARSDEN, H.B., HARRIS, M. & SWINDELL, R. (1984). Excess risk of breast cancer in the mothers of children with soft tisue sarcomas. *Br. J. Cancer*, **49**, 325.

BIRCH, J.M., MANN, J.R., CARTWRIGHT, R.A. & 7 others (1985). The Inter-Regional Epidemiological Study of Childhood Cancer (IRESCC). Study design, control selection and data collection. *Br. J. Cancer*, **52**, 915.

BRESLOW, N.E. & DAY, N.E. (1980). Statistical methods in cancer research. *The Analysis of Case-Control Studies*. **Vol. I:** IARC Scientific Publications no. 32. International Agency for Research on Cancer: Lyon.

DRAPER, G.J., BIRCH, J.M., BITHELL, J.F. & 6 others (1982). Childhood cancer in Britain. HMSO: London.

EPILOG version 2.0 (1985). *Epicentre Software*. PO box 90073, Pasadena, CA 91109.

FRAUMENI, J.F. JR. & BOICE, J.D. (1982). Bone. In *Cancer Epidemiology and Prevention*. Schottenfeld, D. & Fraumeni, J.F. (eds) p. 814. W.B. Saunders Company: Philadelphia.

GRUFFERMAN, S., WANG, H.H., DELONG, E.R., KIMM, S.Y.S., DELZELL, E.S. & FALLETTA, J.M. (1982). Environmental factors in the etiology of rhabdomyosarcoma in childhood. *J. Natl Cancer Inst.*, **68**, 107.

LI, F.P. & FRAUMENI, J.F. JR. (1969). Soft-tissue sarcomas, breast cancer and other neoplasms. A familial syndrome? *Ann. Int. Med.*, **71**, 747.

McKEEN, E.A., HANSON, M.R., MULVIHILL, J.J. & GLAUBIGER, D.L. (1983). Birth defects with Ewing's sarcoma. *N. Engl. J. Med.*, **309**, 1522.

MILLER, R.W. (1981). Contrasting epidemiology of childhood osteosarcoma, Ewing's tumor, and rhabdomyosarcoma. *Natl Cancer Inst. Monogr.*, **56**, 9.

OFFICE OF POPULATION CENSUSES AND SURVEYS. *Classification of Occupations 1970*. HMSO: London.

OPERSKALSKI, E.A., PRESTON-MARTIN, S., HENDERSON, B.E. & VISSCHER, B.R. (1987). A case-control study of osteosarcoma in young persons. *Am. J. Epidemiol.*, **126**, 118.

RUYMANN, F.B., MADDUX, H.R., RAGAB, A. & 5 others (1988). Congenital anomalies associated with rhabdomyosarcoma: An autopsy study of 115 cases. A report from the Intergroup Rhabdomyosarcoma Study Committee (representing the Children's Cancer Study Group, the Pediatric Oncology Group, the United Kingdom Children's Cancer Study Group, and the Pediatric Intergroup Statistical Center). *Med. Pediatr. Oncol.*, **16**, 33.

SPSS-X USER'S GUIDE (1985). McGraw-Hill: New York.

TUCKER, M.A. & FRAUMENI, J.F. JR. (1982). Soft tissue. In *Cancer Epidemiology and Prevention*. Schottenfeld, D. & Fraumeni, J.F. Jr. (eds) p. 827. W.B. Saunders Company: Philadelphia.

WORLD HEALTH ORGANISATION (1978). *International Classification of Diseases*. 9th revision, 1975. WHO: Geneva.